# A general dose-response relationship for chronic chemical and other health stressors and mixtures based on an emergent illness severity model

**DOI:** 10.1371/journal.pone.0211780

**Published:** 2019-02-15

**Authors:** James D. Englehardt, Weihsueh A. Chiu

**Affiliations:** 1 Civil, Architectural, and Environmental Engineering, University of Miami, Coral Gables, Florida, United States of America; 2 Veterinary Integrative Biosciences, Texas A&M University, College Station, Texas, United States of America; Zhongnan University of Economics and Law, CHINA

## Abstract

Current efforts to assess human health response to chemicals based on high-throughput *in vitro* assay data on intra-cellular changes have been hindered for some illnesses by lack of information on higher-level extracellular, inter-organ, and organism-level interactions. However, a dose-response function (DRF), informed by various levels of information including apical health response, can represent a template for convergent top-down, bottom-up analysis. In this paper, a general DRF for chronic chemical and other health stressors and mixtures is derived based on a general first-order model previously derived and demonstrated for illness progression. The derivation accounts for essential autocorrelation among initiating event magnitudes along a toxicological mode of action, typical of complex processes in general, and reveals the inverse relationship between the minimum illness-inducing dose, and the illness severity per unit dose (both variable across a population). The resulting emergent DRF is theoretically scale-inclusive and amenable to low-dose extrapolation. The two-parameter single-toxicant version can be monotonic or sigmoidal, and is demonstrated preferable to traditional models (multistage, lognormal, generalized linear) for the published cancer and non-cancer datasets analyzed: chloroform (induced liver necrosis in female mice); bromate (induced dysplastic focia in male inbred rats); and 2-acetylaminofluorene (induced liver neoplasms and bladder carcinomas in 20,328 female mice). Common- and dissimilar-mode mixture models are demonstrated versus orthogonal data on toluene/benzene mixtures (mortality in Japanese medaka, *Oryzias latipes*, following embryonic exposure). Findings support previous empirical demonstration, and also reveal how a chemical with a typical monotonically-increasing DRF can display a J-shaped DRF when a second, antagonistic common-mode chemical is present. Overall, the general DRF derived here based on an autocorrelated first-order model appears to provide both a strong theoretical/biological basis for, as well as an accurate statistical description of, a diverse, albeit small, sample of observed dose-response data. The further generalizability of this conclusion can be tested in future analyses comparing with traditional modeling approaches across a broader range of datasets.

## Introduction

Identification of chemicals that may pose a health risk following chronic decadal exposure to extremely low doses is a challenge [1], complicated further by possible toxicological interactions among chemicals and other health stressors. Traditional high-dose animal tests have been expensive in terms of time, expense, and animal subjects, may induce extraneous responses such as cytotoxicity, and in any case require extrapolation to low doses of regulatory interest. To address these issues, the U.S. National Research Council [1] recommended assessment based on high-throughput *in vitro* assays targeting intra-cellular processes, and as a result such testing is now producing large databases of high throughput screening (HTS) data [2,3].

One approach proposed for setting regulatory standards based on high throughput screening (HTS) data has been systems biology-based modeling to determine concentrations that would likely lead to excessive perturbation of intracellular pathways, then physiologically-based pharmacokinetic (PBPK) modeling to assess concentrations that would cause adverse effects in humans [4]. However, traditional PBPK models involve extensive efforts to build and validate, typically performed one-chemical-at-a-time. Hence, the need to relate tested concentrations to potential human exposures for thousands of chemicals and assays has led to the development of “high throughput toxicokinetic” approaches, which are implemented as an initial screening approach to identify chemicals with low “margins” between environmental exposures and the exposures that may perturb biological pathways [5,6].

Ultimately, an understanding is needed of the relationship between biological perturbations, including many common stress-response pathways such as oxidative stress response, heat-shock response, and DNA-damage response, and the apical adverse outcomes of interest [4]. While this relationship between perturbation and outcome varies widely among stressor-endpoint pairs, the concept of allostatic load has been used to propose multisystem summary measures of cumulative health stress which have been used to predict health outcomes [7]. Such measures may include, for example, physiological function parameters, including primary mediators in the toxicological cascade, as well as secondary mediators reflecting components of the metabolic syndrome [8].

Both “bottom-up” biologically-based modeling approaches, as well as “top-down” statistical or artificial intelligence-based analyses, have been proposed to discern relationships between collections of related biomarkers, such as changes in gene expression, protein interactions, or metabolite flux, to phenotypic changes within a cell [9–11]. However, truly predictive approaches are still some ways away, particularly for complex effects. For developmental, endocrine, neurotoxicological, and other illnesses, the chronic toxicity of a chemical may depend not only on intracellular pathways, but on causal network dynamics at the extracellular, organ, and organism levels. In that case, information beyond cellular responses to perturbations is needed to assess apical response. As a result, HTS has been little used as yet for chemical regulation [12].

Here, we hypothesize that the lack of a unifying theoretical framework, from cellular perturbation to apical response, is a critical barrier to progress in integrating HTS data into risk assessment. The need for an understanding of the relationship between intra-cellular response, and multi-organ, multi-cellular governing processes at the organism level is recognized, but seems on the face intractable [4]. However, we posit that this relationship is actually reflected in the overall dose-response function (DRF) viewed as a probability distribution on the minimum dose to cause illness in a randomly-selected individual. Thus, a theoretically-derived dose-response functional form can provide a top-down template, and a basis for an analysis in which HTS, multi-tissue co-culture [13], multi-organ chip [14], and animal data can represent prior and posterior information in a Bayesian assessment [15]. Derivation of an appropriate theoretical formulation from which to integrate data at multiple scales can take advantage of convergent top-down, bottom-up analysis, which is being recognized in many fields of natural and social science to have advantages over either approach alone [16,17].

The purpose of this work is to derive and demonstrate a general quantal DRF form for chronic, homogeneously distributed (e.g., not microbial) health stressors, including carcinogenic and noncarcinogenic chemicals, and mixtures thereof. This general DRF is based on an emergent autocorrelated first-order model of illness progression [18], to be described. To derive this model, first, the relationship between the illness severity distribution and the quantal DRF in autocorrelated first-order systems is examined. Then, an emergent first-order multivariate DRF is derived for single stressors, and for mixtures of stressors with common endpoint and accounting for interactions. For illustration, several single chemical dose-response datasets are evaluated using this DRF: chloroform-induced mild cellular liver necrosis in mice [19], 2-acetylaminofluorene-induced liver neoplasms and bladder carcinomas in mice [20], and bromate-induced dysplastic focia in rats [21]. Next, the extension of this approach to multiple stressors is demonstrated using both common- and dissimilar-mode of action mixture models, using an orthogonal dataset on mortality in *Oryzias latipes* due to benzene/toluene mixture exposure [22]. Results are compared with the fit of traditional DRF models, as well as with previously published empirical demonstration [23,24] of the DRF derived theoretically in this work. Applicability to traditional and HTS-based dose-response assessment and extrapolation is also discussed, as well as potential future areas for further demonstration, including analysis of toxic mode of action and other biological aspects.

## Background definitions and methods

Traditional dose-response assessment has involved implicit or explicit extrapolation of response from high testable doses to low, the result of which is determined by the form of the DRF [25], and hence much research has focused on this form [23,26,27]. However, general DRF models, such as the linearized multistage and lognormal models, while relatively flexible, are not considered intrinsically biological in form [28–30], and are theoretically based on the assumption of independence among initiating event magnitudes. In particular, the general form of the linearized multistage model is based on the assumption that “the time from cancer initiation in a single cell until an observable cancer develops in a tissue is … functionally independent of the dose rate” [29], and more generally on the assumption of independent numbers/sizes of initiating events, or *causes*, along a mode of action (MOA) through its basis in the original multistage model [31]. These are important constraints not typically satisfied in complex systems [32]. For example, a cause may be the extent of binding of chromatin modifying complex with histone methyltransferases, which reportedly causes (i.e., is not independent of) chromatin activation and transcriptional activation, which are subsequent causes in an adverse outcome pathway for chemical-induced leukemia [33].

To develop a quantal DRF (i.e. population incidence versus dose), illness must be defined as equal to or greater than a minimum level of illness severity, such as initial malignancy following progressive genetic damage, or non-trivial liver damage. Thus, the form of the population DRF is determined by the distribution of illness severity across the population at a given dose. In other fields of risk analysis, the emergent form of such *incident* size distributions has been observed in complex systems at the macro-level first, and explained mechanistically thereafter. Examples include distributions of disasters and smaller events of the same type, the degrees (number of connections) of nodes in complex dynamical networks, and financial stock return data, all of which are observed to be nearly log-log linear (power laws) across orders of magnitude [34–37]. However, log-log linear probability distributions must be truncated or otherwise terminated at one or both extremes to maintain normalization, and thus represent only a part of the distribution, not extrapolatable beyond the range of available data.

Recently, illnesses and other incidents were argued to arise by a general process in which a series or network of stochastic autocorrelated (i.e., not independent) causes produces illness severities by predominantly first-order (i.e., multiplicative) kinetics, and severities produced as such have emergent Weibull distributions that are characteristically asymptotically log-log linear, as reviewed in the next section. The Weibull distribution spans the non-negative real line, representing the full distribution of outcomes of first-order processes across all physical scales, hence providing a scale-inclusive basis for extrapolation. In particular, the distribution was demonstrated in preference to competing distributions versus available data on cancer and non-cancer illness severity [18,38], as well as other complex system outcomes [35,39,40]. The form was further shown applicable at higher doses, when Michaelis-Menten kinetics begin to apply due to saturation of toxicant receptors.

Some terms are defined as follows. Dose refers to a numerical level of a chronic health stressor, potentially including chemical, economic, environmental, occupational, lifestyle, or other stressors. The size of a cause of an illness is the magnitude of an illness-initiating event, for example the fraction of a toxicant passed to a receptor (not eliminated), or more generally the degree of failure of a protective mechanism. An illness severity distribution is a plot of the population fraction presenting clinical illness, versus a measure of a negative health-effect exceeding the clinical definition of illness, at a given dose (for example, the distribution of observable tumor sizes across a population exposed to a particular common dose), as described in the next section.

Threshold refers to a dose below which no individual responds [41]. The term saturation refers to a dose above which little additional health effect is observed (e.g., above which the toxicant is no longer the rate-limiting reactant). The notations f(.) and F(.) denote continuous probability density function (PDF) and cumulative distribution function (CDF), respectively. Non-scalar (vector, matrix, array) quantities are denoted in bold. Multistage model refers to the one- or two-hit linearized multistage model [29].

Starting with the Weibull illness severity distribution, a new DRF, general to chronic stressors and mixtures thereof, was derived consistent with predominantly first-order kinetics and standard bio-mathematical requirements for DRFs. The derived DRFs were then fitted to published laboratory dose-response data by minimization of the deviance statistic, Y*, an adaptation of the 2-log-likelihood ratio to quantal dose-response data [42]. Fits were evaluated by inspection, and compared with the fit of the multistage, lognormal (*μ*, *σ*), and generalized linear models as appropriate.

Following visual inspection, fitted DRFs were further appraised for goodness-of-fit (GOF) based on their *p*-value. This value was obtained as the value of the chi-squared CDF with *I* − *m* − 1 degrees of freedom, at Y*, a strict test in which *I* is the number of doses tested, *m* is the number of parameters in the fitted distribution, and the degrees of freedom are decremented by unity to account for the assumption of a particular parametric form. The approach is rigorous and asymptotically-equivalent to the chi-square test [43], though data at doses of zero and at doses for which either zero positive or zero negative subjects are observed, cannot be used (due to required logarithmic calculations). Hence, except in analysis of the benzene-toluene mixture data analyzed and reported previously, the plotting position *n* = Min[Max(0.25, *n*), *N*-0.25], in which *n* is the number of positives and *N* is the total number of subjects, was used. For example, for *N* = 10, values of *n* = 0 would be set equal to 0.25, and values of *n* = 10 would be set equal to 9.25. In contrast with other proposed plotting positions [44], this approach allows use for GOF analysis of the information that < 1 responder (non-responder, for *n* = *N*) in *N* individuals was observed at particular doses, by accounting for the finite binomial probability of observing ≥ 1 responder (non-responder) if the sample size had been larger.

**Fig 1:**
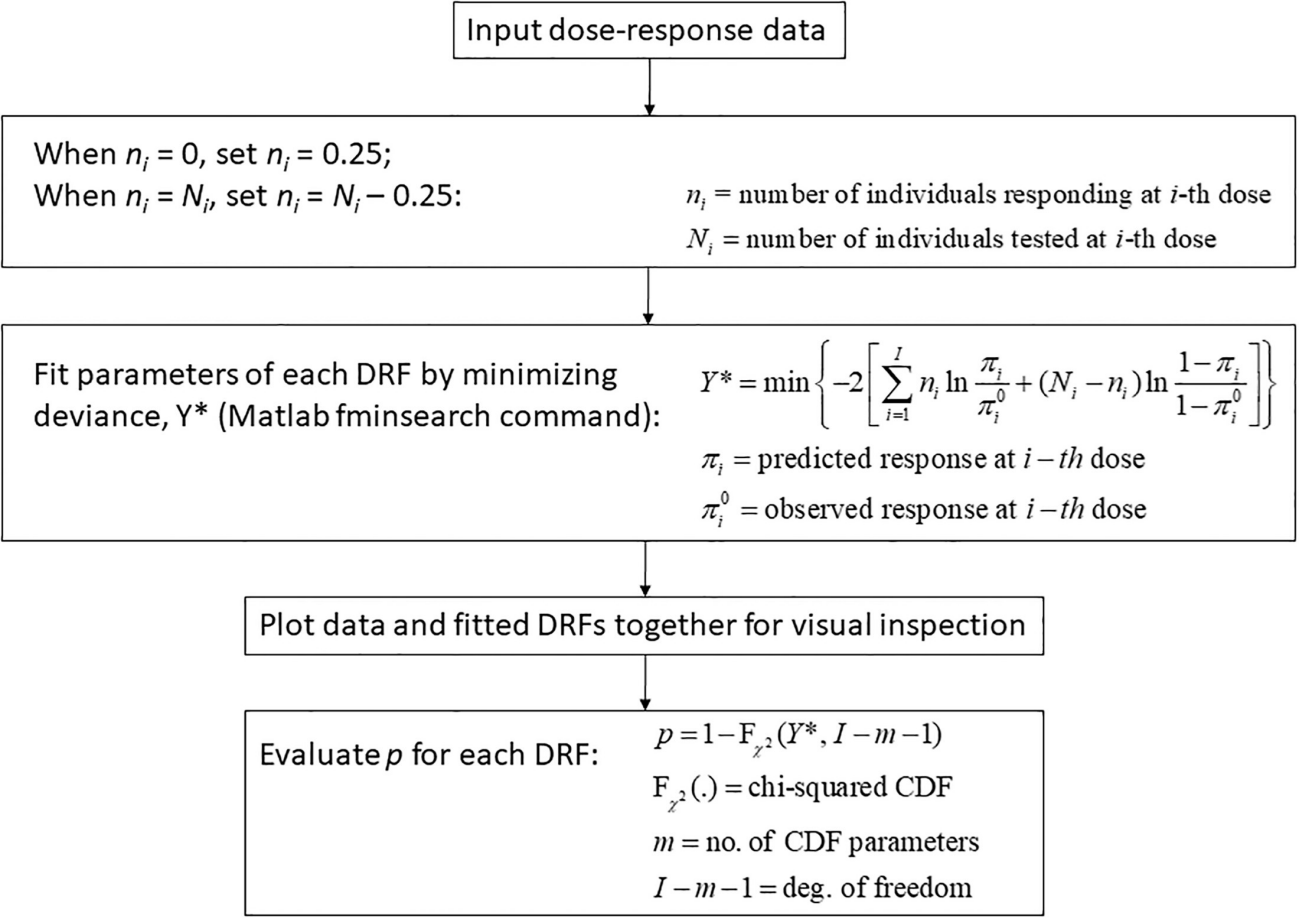
Diagram illustrating the procedure for data processing.

The data processing procedure just described is diagrammed in Fig 1. GOF tests were coded in Matlab version R2006a with Statistics Toolbox. Results for all datasets tested are reported herein, except as noted or when found insufficient for evaluation.

## Dose-response model derivation

### Relationship of illness severity distributions to the quantal DRF

**Fig 2:**
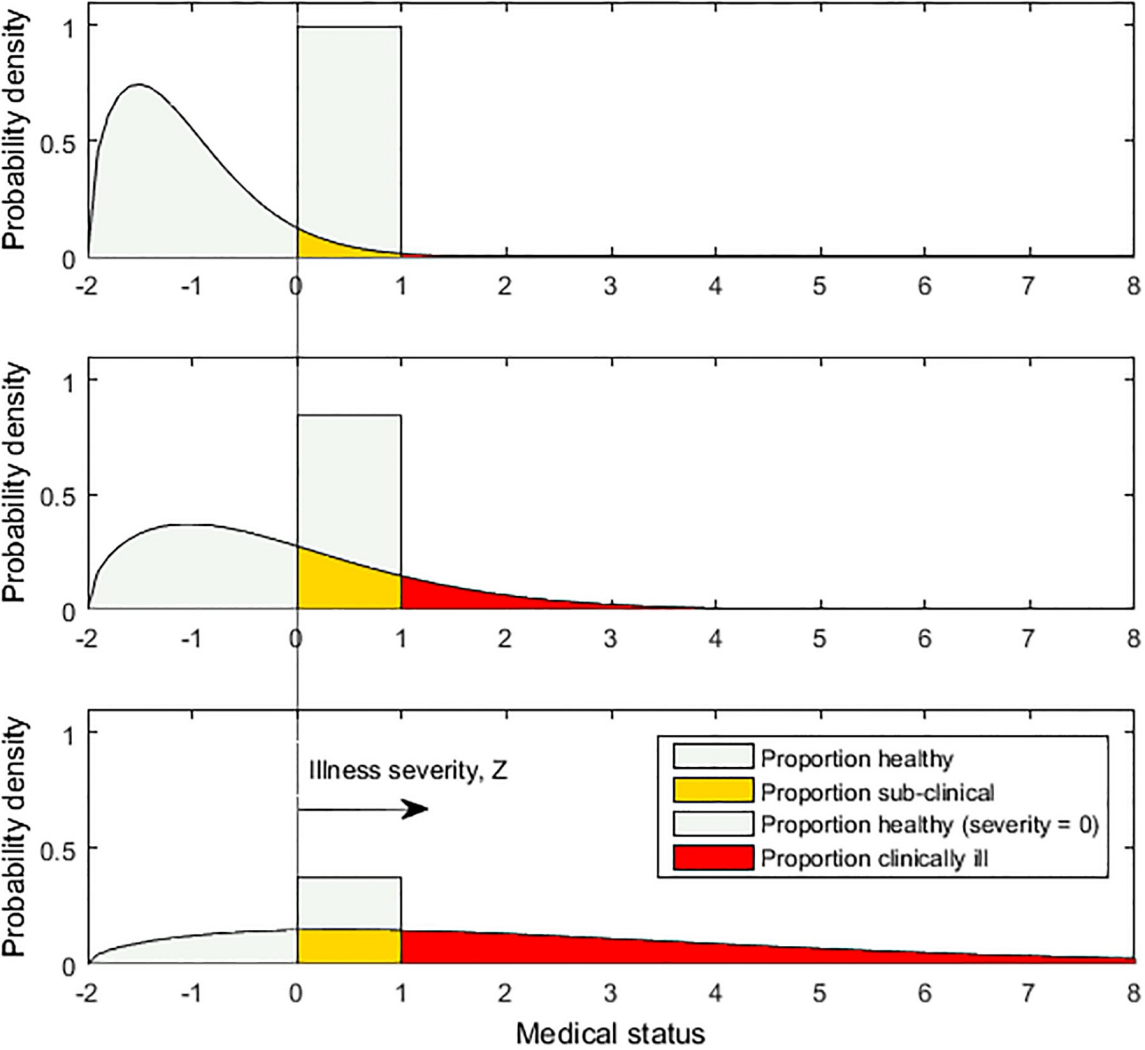
Diagram illustrating hypothetical illness severity distributions for three arbitrary doses, increasing from top panel to bottom panel. The three PDFs comprise distributions of numerical medical status (e.g., blood pressure), censored at the clinically-defined lower bound of health impairment, and thus having a monotonically-decreasing shape. The value of population response that would be indicated on a DRF, for a given dose, is the area (red or dark grey) under the illness severity distribution for that dose, integrated over the range of illness severity considered to represent the desired illness endpoint.

To derive a quantal DRF for chronic health stressors, the relationships between dose, illness severity, and resulting “response,” or fraction of the population becoming ill, can be examined as illustrated in Fig 2. As shown, at each constant dose administered to a randomly-selected subpopulation, the subpopulation presents a continuous (Weibull) distribution (PDF) of “medical status,” with status deteriorating towards the right. The higher the dose, the longer is the tail of the distribution representing individuals responding severely. (Note that illness severities are assumed sampled at a fixed time after exposure, and measured in terms of their magnitude at that time. For example, cancer progresses through increasing stages of genetic damage, malignancy, and growth, such that severity increases with time and hence population severity distributions vary with time).

As shown in Fig 2, in general, the medical status distribution can be divided into three ranges for purposes of constructing a quantal dose-response function, though not all may be observed for a given stressor: (a) high wellness or health (light green or lightly shaded/white), e.g. blood pressure or genes representing the normal condition, below the “tipping point” at which irreversible changes in medical status occur [45], (b) moderate wellness or sub-clinical effect (yellow or grey), e.g. effects include at least pre-hypertension/hypertension or increasing genetic damage but no malignant cell, and (c) low wellness or clinically-defined illness (red or dark grey), e.g. effects include at least hypertension/minor cardiovascular event (minor stroke, heart attack) or one or more malignant cells. Some healthy individuals may even experience a health benefit (hormesis) from a low dose, e.g. increased immunity, though this condition would generally represent a different endpoint not depicted in the severity distribution, and in any case would not affect the corresponding quantal dose-response function for illness.

To develop a distribution of medical status across the population at fixed dose, status must be characterized numerically, e.g. in units of blood pressure. Accordingly, the value of medical status considered clinically to be the lower bound of health impairment is designated for discussion as *Z* = 0, and the value considered clinically to be the lower bound of illness is designated similarly as *Z* = Z* = 1, in arbitrary units. Then, superimposed on the medical status distribution is the illness severity distribution, which is the medical status distribution left-censored at *Z* = 0. That is, healthy individuals (medical status less than zero) are included with those having health impairment below clinically-defined illness (0 ≤ Z < 1), to make up the total proportion of individuals not presenting illness. Hence, as shown in Fig 2, illness severity PDFs generally decrease monotonically, from a high proportion of individuals who are not ill, towards increasing illness, defined as significant severity. Similarly, causes of illness and incidents also must be large (severe) enough to impact an illness outcome, and therefore the censored PDFs of cause size are also monotonic. Ultimately, the population response that would be associated with a particular dose on a quantal dose-response curve is shown to be the area under the illness severity curve for that dose, from Z = Z* to ∞.

Each successive cause of an illness, like causes of other seemingly complex systems, typically affects the size, or severity, of the final illness in proportion to its own size. Hence, if all cause sizes are fixed, illness severity is largely proportional to the product of the cause sizes. An analogy is the multiplicative effect of interest rates on the future value of an investment. For example in terms of toxicology, the concentration of a toxicant at a target organ results as the product of fractions (percents) of the toxicant passed at a series of organs along an MOA. Likewise, illness severity may result as the product of the rates that mutations in oncogenic or tumor suppression genes escape cell growth controls and correction processes, such as DNA repair mechanisms or apoptosis, at successive steps along an MOA.

Because cause sizes vary, each having a monotonically-decreasing distribution across a population, the resulting distribution of products will comprise vastly more healthy individuals and vastly fewer extremely ill individuals. Further, if such cause sizes were independent of each other, then the result would be an asymptotic lognormal distribution of illness severity, by the Central Limit Theorem (sums of logs representing logs of products of random variables are normally distributed). However, cause sizes in real systems are generally correlated in size [18,32]. Correlation is due either to a common cause, such as aging or organ (e.g. brain) damage impacting multiple functions, and/or to the fact that larger upstream failures in physiological defensive mechanisms stress downstream mechanisms more heavily, potentially precipitating larger downstream failures. This correlation skews the resulting distribution further, with the result being an emergent Weibull distribution of illness severities across a population [18], by transformation of variables and the tendency of physical systems towards the maximum entropy (most likely) distribution [46].

Recognition of the Weibull distribution of illness severities resolves several practical issues. First, unlike power law distributions, the distribution is scale-inclusive, extending across the full range of observed severities. Also, as expected for outcomes such as illness severity, the Weibull PDF can be monotonic, in contrast with the lognormal PDF which would indicate essentially zero probability of essentially no illness, and can apply over orders of magnitude. The distribution likewise describes medical status at intermediate points along the MOA. For example, in a population exposed simultaneously, but not yet fully presenting illness, a range of blood pressures, or a range in the extent of genetic transcription errors, may be observed.

The multiplicative, or first-order, model can be represented in discrete form as $Z=C_{0}C_{1}C_{2}•••C_{t}=C_{0}\prod_{i=1}^{t}C_{i}$, in which *Z* is illness severity; *C*_*0*_ is the dose, ranging from threshold to total saturation; the remaining *C*_*i*_ are the *i*-th succeeding random cause sizes, ranging from ∞ to -∞ (causes can decrease severity); and *t* indicates the total number of increments along an MOA (analogous to the number of time periods of financial compounding) [18]. Note that while the dose may not be random in general, dose is generally autocorrelated with succeeding cause sizes, over a range of doses. (The discrete form is functionally equivalent to the continuous form, $Z=C_{0}\prod_{t=1}^{T}C_{t}\equiv C_{0}\prod_{t=1}^{T}\left(1+r_{t}\right)\;\underset{\Delta t\to 0}{\to }\;C_{0}\pi_{t=0}^{T}e^{rdt}$, in which the *r*_*t*_ are random dependent first-order rate constants, analogous to interest rates in time; the $e^{r_{t}}$ represent vanishingly-small autocorrelated random cause sizes; *r* is a continuously-varying first-order rate constant approached in the limit as Δ*t*→0; and $\pi_{t=0}^{T}e^{rdt}$ is a product integral [18,32]. Also, in a first-order model, the *C*_*i*_ can be expressed in any units without affecting the form of the distribution of their product).

### Emergent first-order single-stressor model

To derive the (quantal) DRF, let *Z* = *D•W*, in which *D* is the *critical* dose, or minimum dose to cause illness in a randomly-selected individual, a random variable with CDF equivalent in essence to the DRF, also interpreted as the (minimum) size of the initial cause of illness; and *W* is severity per unit dose, a random variable also interpreted as the product of the sizes of causes subsequent to dose absorption. Across a population of individuals with varying susceptibility as indicated by their critical dose, *D*, all three variables can be random and correlated for a general exposure scenario, and hence linearity of response, *Z*, with dose, *D*, is not implied. Nevertheless, at any given dose, the distribution of subsequent physiological response, *W*, across a population defines the fraction of individuals responding at a particular severity level, *Z*. Consequently, for a fixed clinical definition of illness corresponding to a minimum severity, *Z* ≥ Z*, the form of the physiologically-based distribution of *W* defines the form of the physiologically-based distribution of *D* across a population.

Letting the assumed clinical definition of illness be *Z* ≥ Z* = 1, in arbitrary units, the critical dose can be written *D* = Z*/*W* = 1/*W*. This inverse relationship between severity per unit dose and the critical dose, for a constant severity, is expected because individuals who present a high severity per unit dose (therefore being represented in the upper tail of the severity distribution) will have a low critical dose (i.e. will respond at the low-dose end of the DRF). Letting *W*, like *Z*, be distributed Weibull [18], then *D* is proposed to be necessarily distributed Fréchet by transformation of variables [47]. That is, the probability of response at a dose is $\text{F(}d)=\text{exp}\left[-{\left(d/\xi \right)}^{-\eta }\right]$, in which *η* is a positive shape parameter, and *ξ* is a positive scale parameter. In physical systems, the exponential parameter *η* reflects the number of first-order compounding increments, and the degree of autocorrelation. Overall, the lumped parameter may be viewed as a logarithmic-scale parameter [18].

The Fréchet distribution is the first-order DRF accounting for response to single stressors above background. For chemicals and other stressors having a threshold dose, *d*_*0*_, the shifted, three-parameter Fréchet can be written:

$$\begin{array}{l} F_{F}(d)=\text{exp}\left\{-{\left[(d-d_{0})/\xi \right]}^{-\eta }\right\}\\ F_{F}(d\prime )=\text{exp}\left\{-{\left[d\prime \right]}^{-\eta }\right\},\;\;d,d_{0},d\prime \geq 0. \end{array}$$ (1)

in which *d'* is a scaled dose above threshold. Of note, the Fréchet can be monotonic in shape, like the single-hit multistage model and as may be expected for stressors having no threshold dose, or sigmoidal in shape, like the two-hit multistage model and as might obtain for any stressor.

### Common mode mixture model

When components of a chemical mixture act by a common toxicological mode of action, illness response is considered to be a function of the sum of the individual doses, scaled by their relative toxicities [27]. Then, when population response is greater/less than that predicted by such dose addition, synergism/antagonism is indicated. In this work, the concept of dose addition is generalized, such that the total dose may also include additive positive or negative terms of the same order, accounting for synergistic or antagonistic biological interactions among those stressors. Such terms can be viewed as additional doses, positive or negative, acting by the same common mode and thus similarly additive. Hence, neglecting three-way and higher order interactions, all (two-way) interaction terms should be (a) additive, (b) accounting for both doses while of the same order as individual doses, and (c) naught when either dose is zero.

To develop a general common-mode mixture DRF, accounting for cases in which interactions are not observed at low doses [48,49], all doses and interactions should be subject to potential *interaction thresholds* below which the interaction is not observed (though some thresholds may appropriately be set at zero, e.g. for cancer). Further, all common-mode stressors should share toxicological causes, generally common in number, log-scale, and autocorrelation, and therefore a common value of 1/*η* by first-order theory [18]. Finally, the function, when considered a multivariate CDF, should not have Fréchet marginals, otherwise predicted response would be zero when the dose of any component in the mixture was zero. Rather, the univariate function obtaining in each dimension when all other doses equal zero, and thus representing the cumulative distribution given all other doses equal to zero, must be Fréchet.

The simplest common-mode mixture DRF meeting the criteria just outlined is proposed as:

$$F_{\text{CM}}(d)=\text{exp}\left\{-\left[{\left(\text{max}\left\{\left[\left(\sum_{j}\frac{d_{j}}{\xi_{j}}\right)+\left(\sum_{j\neq k}\xi_{j,k}\sqrt{d_{j}d_{k}}\right)-d_{0}\right],\;0\right\}\right)}^{-\eta }\right]\right\}$$ (2)

In Eq 2, **d** is a vector of *J* positive real doses, *d*_*j*_, of different stressors; F_CM_(**d**) is the probability of response in terms of a common illness endpoint; *ξ*_j_ is the positive real scale parameter of the *j*-th chemical or stressor; *ξ*_*j*,*k*_ = *ξ*_*k*,*j*_ are real scale parameters for the interaction between the *i*-th and *j*-th chemicals or stressors, equal to zero when no interaction occurs; and *η* is a positive real shape parameter representing the number, log-scale, and autocorrelation of illness causes, common across components of the mixture.

Eq 2 is closed-form, and ensures zero response below threshold via the maximum operator (when *d*_*0*_ ≠ 0). The geometric mean interaction terms are zero when a constituent dose is zero, and otherwise represent interactions on the same order as those of the individual doses, like the covariance terms of the multivariate normal distribution. The DRF can be further generalized to include three-way and higher-order interactions similarly, though at considerable expense in terms of parameterization and data demand. These interaction “dose” terms are all assumed subject to threshold values, or interaction thresholds, below which no interaction is observed. Because scaled doses and interaction terms are additive, and all corresponding thresholds are constant, all thresholds can be expressed by a single constant. Accordingly, *d*_*0*_ is a constant representing the overall scaled threshold dose for the total scaled dose of a mixture.

### Dissimilar mode and general mixture model

Dissimilar-mode health stressors, acting along parallel causal pathways, or MOAs, to a common midpoint or endpoint, have largely independent probabilities of causing illness (e.g., for a given dose to a randomly-selected human subject, defensive mechanisms of one pathway acts largely independently of those of other pathways). Therefore, the probability that the critical total dose, **D**, of a mixture is less than or equal to some dose, **d**, corresponds to the union of the events, *D*_*i*_ ≤ *d*_*i*_, that any individual stressor causes illness. Then the probability of illness can be found by the inclusion–exclusion principle as the probability of the union of the events that doses *d*_*1*_, *d*_*2*_, … *d*_*I*_ cause illness:

$$\text{F}(d)=\sum_{i}{\text{F}}_{\text{F}}(d_{i})-\sum_{i\neq k}{\text{F}}_{\text{MF}}\left(d_{i},d_{k}\right)+\sum_{i\neq k\neq l}{\text{F}}_{\text{MF}}\left(d_{i},d_{k},d_{l}\right)-\ldots \pm {\text{F}}_{\text{MF}}\left(d_{1},d_{2},\ldots ,d_{I}\right)$$ (3)

in which F_MF_(*d*_*i*_, …) is the joint probability that the set of doses (*d*_*i*_, …) is collectively at least great enough to cause illness.

Eq 3 can be rewritten, as:

$$\text{F}(d\prime )=\sum_{i}{\text{F}}_{\text{F}}(d\prime_{i})-\sum_{i\neq k}{\text{F}}_{\text{MF}}\left(d\prime_{i},d\prime_{k}\right)+\sum_{i\neq k\neq l}{\text{F}}_{\text{MF}}\left(d\prime_{i},d\prime_{k},d\prime_{l}\right)-\ldots \pm {\text{F}}_{\text{MF}}\left(d\prime_{1},d\prime_{2},\ldots ,d\prime_{I}\right)$$ (4)

in which **d'** is the vector of scaled effective doses accounting for interactions.

Then a new set of scaled effective doses, adjusted for all two-way interactions (and generalizable similarly to account for higher order interactions), is proposed as follows:

$$d\prime_{i}=\text{max}\left[\frac{\text{max}\left(d_{i}-d_{0,i},\;0\right)}{\xi_{i}}+\sum_{k\neq i}\xi_{i,k}\text{max}\left(\sqrt{d_{i}d_{k}}-d_{0i,k},\;0\right),\;0\right]$$ (5)

in which the *d*_*0 i*,*k*_ are interaction-specific threshold doses for each of the $\sum_{i=1}^{I}(i-1)$ pairs of dissimilar-mode stressor doses, (*d*_*i*_, *d*_*k*_), and *I* is the number of individual health stressors. Thus, recognizing their relatively smaller contribution, interactions are represented as additive adjustments to the effective dose of each individual stressor, rather than as separate stressors with modes of action (and *η*) distinct from those of the interacting stressors. Also, distinct thresholds are provided for each interaction in Eq 4 to maintain generality, e.g. to allow for nonzero thresholds below individual stressor thresholds as a result of synergistic interactions.

In Eq 4, the F_MF_(.) terms are typically small relative to F(.) terms, and often neglected [27]. Alternatively, the doses of dissimilar-mode mixture components, following their adjustment to account for interactions by Eq 5, may reasonably be assumed to act independently. That is, the events that doses *d'*_*1*_, *d'*_*2*_, …, *d'*_*I*_ cause illness are essentially independent. Thus, the probability, F_MF_, that the doses of more than one stressor in the mixture are sufficient to cause illness is seen to be:

$${\text{F}}_{\text{MF}}(d\prime_{1},\;d\prime_{2},\ldots )=\prod_{i}{\text{F}}_{\text{F}}(d\prime_{i})$$ (6)

Eqs 4–6 represent a generalization of the concept of response addition, to allow dose adjustment for interactions prior to the assessment of response based on the formula for the probability of the union of independent events [27]. Note that, in Eqs 4–6, each *i*-th stressor is assigned a distinct *η*_*i*_, to model dissimilar causal pathways, in contrast with the common mode model. Similarly, distinct *d*_*0 i*,*k*_ are provided for the interactions. Thus, for example, the dissimilar-mode model for a two-stressor mixture is [23]:

$$\text{F}(d\prime )={\text{F}}_{\text{F}}\left(d\prime_{1}\right)+{\text{F}}_{\text{F}}\left(d\prime_{2}\right)-{\text{F}}_{\text{F}}\left(d\prime_{1}\right){\text{F}}_{\text{F}}\left(d\prime_{2}\right)$$

$${\text{F}}_{F}(d\prime_{1})=\text{exp}\left\{-\text{max}{\left[\frac{\text{max}\left(d_{1}-d_{0,1},\;0\right)}{\xi_{1}}+\xi_{1,2}\text{max}\left(\sqrt{d_{1}d_{2}}-d_{01,2},\;0\right),\;0\right]}^{-\eta_{1}}\right\}$$

$${\text{F}}_{F}(d\prime_{2})=\text{exp}\left\{-\text{max}{\left[\frac{\text{max}\left(d_{2}-d_{0,2},\;0\right)}{\xi_{2}}+\xi_{1,2}\text{max}\left(\sqrt{d_{1}d_{2}}-d_{01,2},\;0\right),\;0\right]}^{-\eta_{2}}\right\}$$ (7)

Eq 4 can be considered a general DRF for health stressors having a common endpoint, regardless of MOA, as follows. First, Eqs 4–6 represent a DRF for strictly dissimilar-mode stressors, acting either independently or with interaction. Also, Eq 4 holds for the union of any events, regardless of independence or MOA, and hence represents a theoretical generalization of Eq 2. Therefore, Eqs 4–6 can model partially-dissimilar-mode stressors acting by dissimilar modes to an intermediate point followed by a common mode to a single endpoint. Further, Eqs 4–6 can also give the probability of response resulting from any mixture of chronic common- and dissimilar-mode stressors. That is, any component of the mixture, **d**, might itself be a mixture of stressors acting by a single common mode, distinct from the modes of remaining components of the mixture. Accordingly, any of the *d*_*i*_ in Eq 4 may represent a total dose of stressors acting by a distinct but internally-common mode of action, transformed consistent with Eq 2 as equal to:

$$d_{i}=\text{max}\left\{\sum_{j}\frac{d_{j}}{\xi_{j}}+\sum_{j\neq k}\frac{\sqrt{d_{i}d_{k}}}{\xi_{j,k}}-d_{0i},\;0\right\}$$ (8)

### Representing background risk

Unidentified background stressors typically result in a minor rate of illness in the unexposed population, which affects the dose-response analysis. For carcinogens, the background risk is represented by the parameter *q*_*0*_ in the multistage model, F_M_ = 1 − exp(-*q*_*0*_ − *q*_*1*_*d* − … − *q*_*k*_*d*^k^), in which *q*_*0*_, *q*_*1*_, …, *q*_*k*_ are non-negative parameters and F_M_ is the total response. For non-carcinogens, a common approach has been to apply the transformation DRF = *c* + (1-*c*) × CDF [50], in which *c* is a parameter added to represent the background risk in terms of response. Mathematically, this technique amounts to response addition with renormalization, implying a different mode of toxic action by the background stressors relative to the stressor being analyzed, as may often occur, and this approach can be used with the first-order model. Alternatively, a common toxic mode can be assumed for the background stress, by adding a parameter, *d*_*b*_, representing a “dose” of total endpoint-specific background stress. That is, Eq 1 can be written F_F_(*d*) = exp{-[*d*_*b*_ + (*d* − *d*_*0*_)/ *ξ*]^-*η*^}. Similarly, a background stress, *d*_*b*_, can be added to the common-mode mixture model of Eq 2. In the same way, a parameter *d'*_*b*_ = (*d*_b_ − *d*_*0* b_)/ *ξ*_b_, again representing background stress, could be added to any of the other doses, *d*_*i*_, in Eq 5, for example to study interactions of background stress with individual dissimilar-mode stressors.

## Empirical evaluation

**Fig 3:**
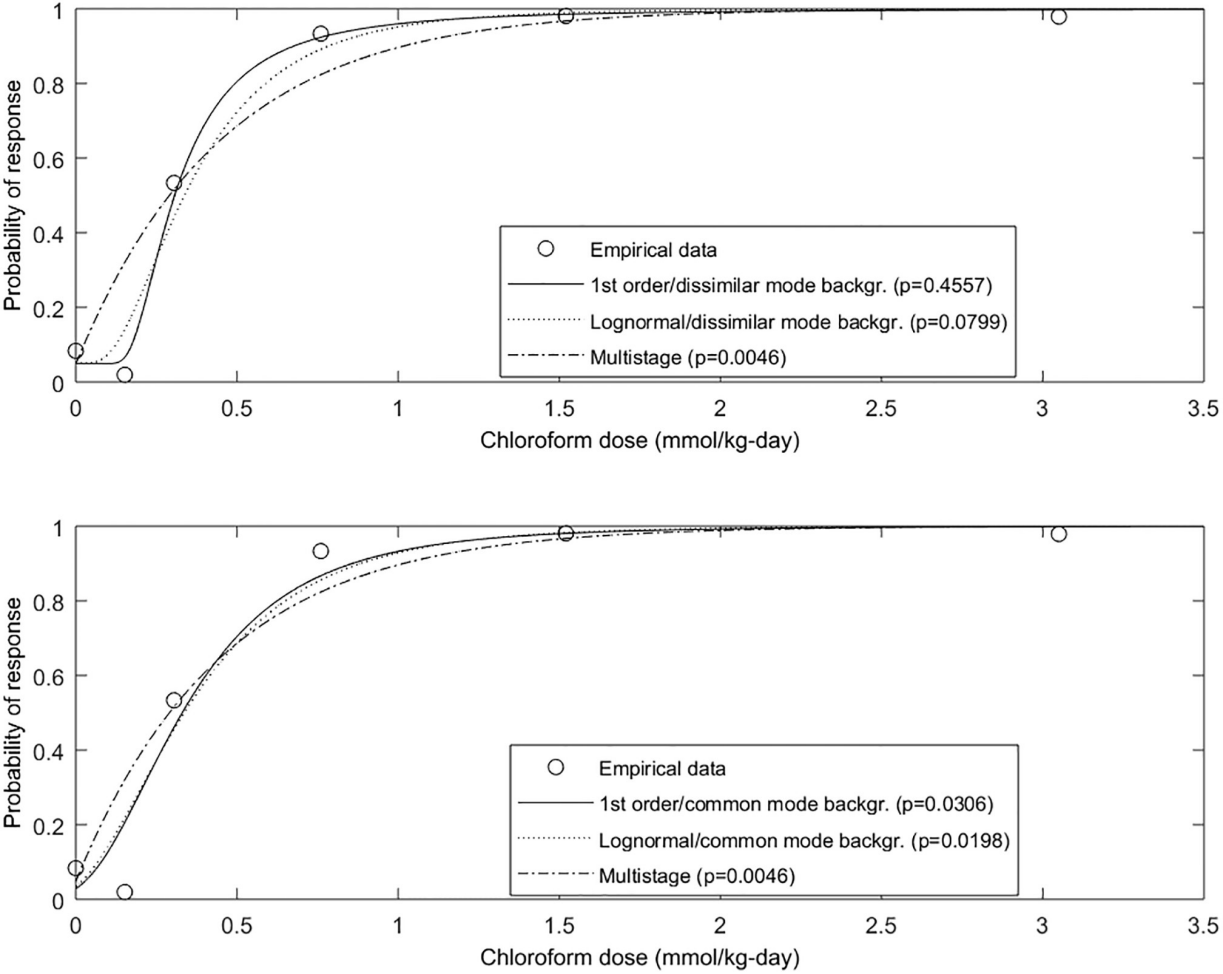
First-order/dissimilar-mode background (DMB), first-order/common-mode background (CMB), multi-stage, and lognormal DRFs versus data on chloroform-induced mild cellular liver necrosis in mice.

The emergent first-order DRF of Eq 1 was first compared with observed mild cellular liver necrosis (any observed necrotic hepatocytes seen) in female CD-1 mice following 14 day exposure to orally-administered chloroform [19] (S1 Table). Because of the observed response at zero dose, these data were used to compare the approaches just described for handling background risk, assuming both common and dissimilar mode background stress. First, the first-order model, transformed as F_F,DMB_ = *c* + (1-*c*) × F_F_, was fitted and compared with the lognormal transformed similarly and the multistage model. Then, observed liver damage was considered to result from a binary mixture of chloroform, modeled with scale parameter *ξ*_*1*_, and “dose” of background liver stress, *d*_*b*_, acting by common mode with no interaction. Again because a response was observed at dose zero, a threshold dose was not observable and was assumed conservatively at zero. Thus, the first-order common-mode mixture model F_F_(*d*) = exp{-[*d*_*b*_ + *d*/ *ξ*_*1*_]^-*η*^} was fitted to the data, and compared with the lognormal, shifted likewise with the addition of a parameter, *d*_*b*_, and the multistage model. As shown in Fig 3 and Table 1, the first-order model was associated with the highest *p*-value, suggesting better fit (though *p*-values are not strictly comparable across models), particularly when assuming a dissimilar-mode background (DMB) stress (*p* = 0.4557), though the lognormal with dissimilar mode background also passed (*p* = 0.0799).

**Table 1:** Empirical GOF results for all datasets.

Chemical/endpoint	DRF Model	*χ2* *p*-value	Parameters
Chloroform/ mild cellular liver necrosis	First-order DMB	0.4557	*c* = 0.0496, *ξ*_1_ = 0.2719, *η* = 2.4126
	Lognormal DMB	0.0799	*c* = 0.0507, *μ* = -1.0434, *σ* = 0.6393
	First-order CMB	0.0306	*d*_*b*_ = 0. 7886, *ξ*_1_ = 1.1711, *η* = 5.3731
	Lognormal CMB	0.0198	*c* = 0.1896, *μ* = -0.6417, *σ* = 0.5555
	Multistage	0.0046	*q*_*0*_ = 0.0486, *q*_*1*_ = 2.2191, *q*_*2*_ = 0
Bromate/ dysplastic focia	First-order CMB	0.1473	*d*_*b*_ = 0.5485, *ξ*_*1*_ = 7.0161, *η* = 2.1617
	First-order DMB	0.0434	*c* = 0.0243, *ξ*_*1*_ = 2.6715, *η* = 1.0072
	Multistage	0.0905	*q*_*0*_ = 0.0113, *q*_*1*_ = 0.1367, *q*_*2*_ = 3.0472e-8
2-acetylaminofluorene/liver neoplasms 18 mo.	First-order CMB	0.5894	*d*_*b*_ = 0.7015, *ξ*_*1*_ = 1025.7, *η* = 4.4386
	First-order DMB	0.8116	*c* = 0.0116, *ξ*_*1*_ = 653.1273, *η* = 0.5455
	Multistage	0.6318	*q*_*0*_ = 0.0119, *q*_*1*_ = 1.0133e-7, *q*_*2*_ = 5.9001e-6
2-acetylaminofluorene/liver neoplasms 33 mo.	First-order CMB	2.5134e-011	*d*_*b*_ = 0.7023, *ξ*_*1*_ = 106.6296, *η* = 1.6303
	First-order DMB	2.7750e-011	*c* = 0. 1676, *ξ*_*1*_ = 50.3095, *η* = 0.7745
	Multistage	1.2779e-013	*q*_*0*_ = 0.3378, *q*_*1*_ = 9.6018e-8, *q*_*2*_ = 8.6465e-5
2-acetylaminofluorene/bladder carcinomas 18 mo.	First-order CMB	0	*d*_*b*_ = 0.0070, *ξ*_*1*_ = 135.1038, *η* = 3. 8718*
	First-order DMB	0.1827	*c* = 0.0070, *ξ*_*1*_ = 135.1038, *η* = 3.8718
	Multistage	0	*q*_*0*_ = 7.1776e-4, *q*_*1*_ = 8.5288e-8, *q*_*2*_ = 1.1109e-5
2-acetylaminofluorene/bladder carcinomas 33 mo.	First-order CMB	0	*d*_*b*_ = 0.25, *ξ*_*1*_ = 110, *η* = 7*
	First-order DMB	0.3304	*c* = 0.0106, *ξ*_*1*_ = 84. 0961, *η* = 5.1121
	Multistage	0	*q*_*0*_ = 0, *q*_*1*_ = 1e-20, *q*_*2*_ = 0.00008*
Benzene-toluene/mortality	First-order CM	0.2825	*ξ*_1_ = 3, *ξ*_2_ = 30, *ξ*_1,2_ = -0.095 *η* = 1, *d*_*0*_ = 4.2
	First-order DM	0.7804	*ξ*_1_ = 6, *ξ*_2_ = 30, *ξ*_1,2_ = 0, *η*_*1*_ = 1, *η*_*2*_ = 1, *d*_*0* 1_ = 10, *d*_*0* 2_ = 125, *d*_*0* 1,2_ = 0

*Parameters selected manually based on substantially improved visual fit.

**Fig 4:**
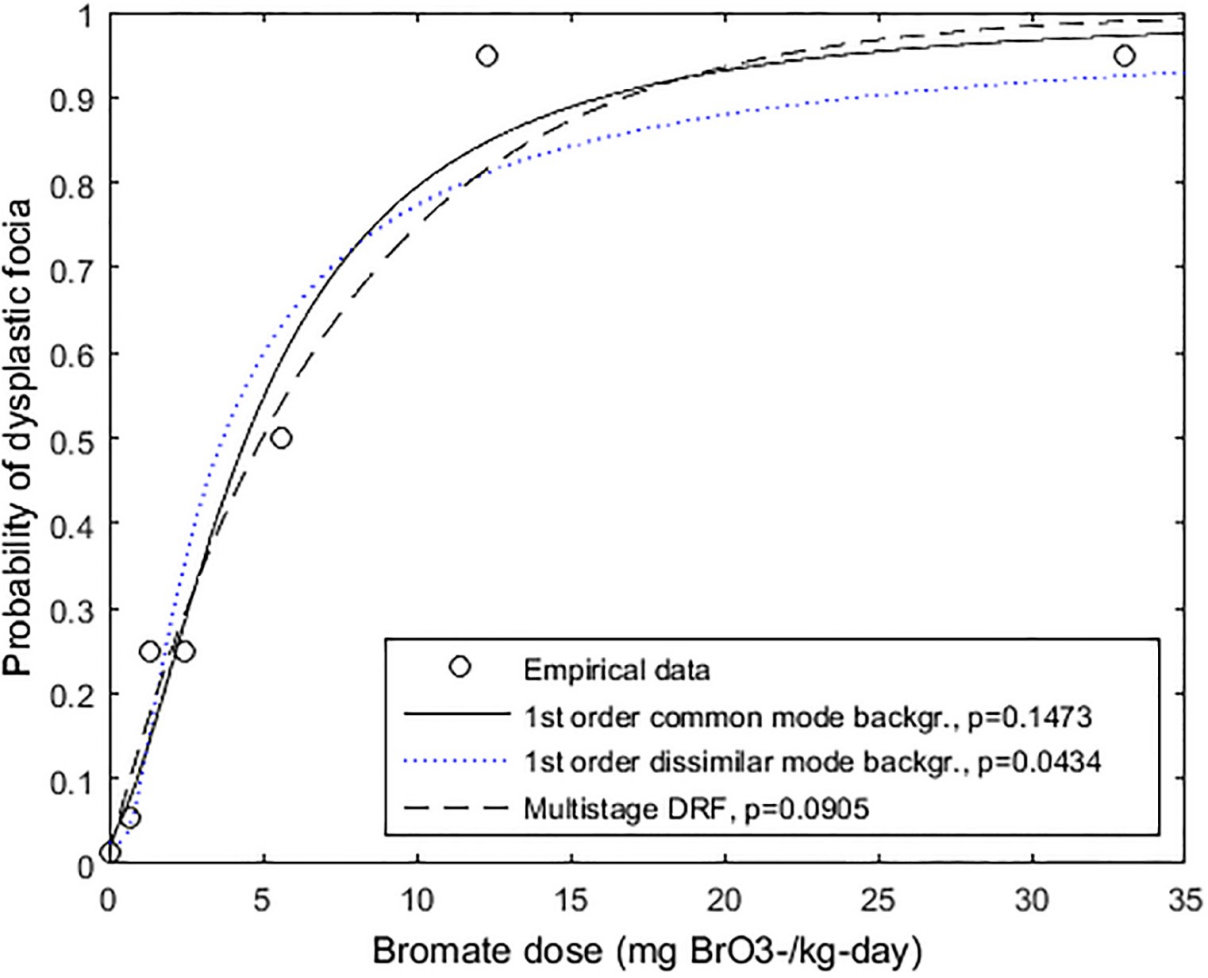
First-order/dissimilar-mode background (DMB), first-order/common-mode background (CMB), and multi-stage DRFs versus data on bromate-induced dysplastic focia (reported pre-neoplastic) in male inbred F344 rats.

The first-order model was also compared to the multistage model versus data on chronic toxicity of aqueous potassium bromate to groups of 20–24 male inbred F344 rats for 104 weeks (S1 Table) [21]. Data for the endpoint dysplastic focia, considered by the researchers to be a preneoplastic lesion, represented sufficient range and resolution for the dose-response analysis, and therefore were used. The first-order non-threshold model with DMB and common-mode background (CMB) stress with no interaction were considered, and compared with the multistage cancer model. As shown in Fig 4 and Table 1, not all animals responded at the two highest doses, suggesting the possibility of a fraction of barely susceptible individuals in the population. As shown, the first-order model assuming CMB stress was associated with the highest *p*-value (*p* = 0.1473), particularly in modeling the barely-susceptible segment.

Though few chemical dose-response datasets are rich enough for significant and general model comparison, the data on liver neoplasms and bladder carcinomas of the ED_01_ study [20] include seven non-zero dose/response pairs for which response was regularly above background, representing no fewer than 20,328 BALB/c female mice fed chronic doses of 2-acetylaminofluorene, including those dying between preselected sacrifice intervals, analyzed for several endpoints (S2 Table). Data on two endpoints, liver neoplasm and bladder carcinoma, were sufficient and were used to evaluate the first-order (non-threshold) model for carcinogens. Because US human life expectancy was 79.8 y over the period 2010–2013, and the standard lifetime exposure factor for risk analyses is 70 y, and BALB/c female mouse intermediate lifespan is 20 months [51], data for each endpoint at (70/80) x 20 = 18 months were used. In addition, data at 33 months for each endpoint were analyzed for comparison with time-to-tumor analysis of this dataset presented previously [18]. Because a background rate of illness was apparent (all datasets have non-zero *y*-intercepts), the same models incorporating background stress as shown in Fig 4 were compared for these cancer data. Empirical DRFs were plotted versus the first-order CMB, first-order DMB, and multistage cancer DRF models.

**Fig 5:**
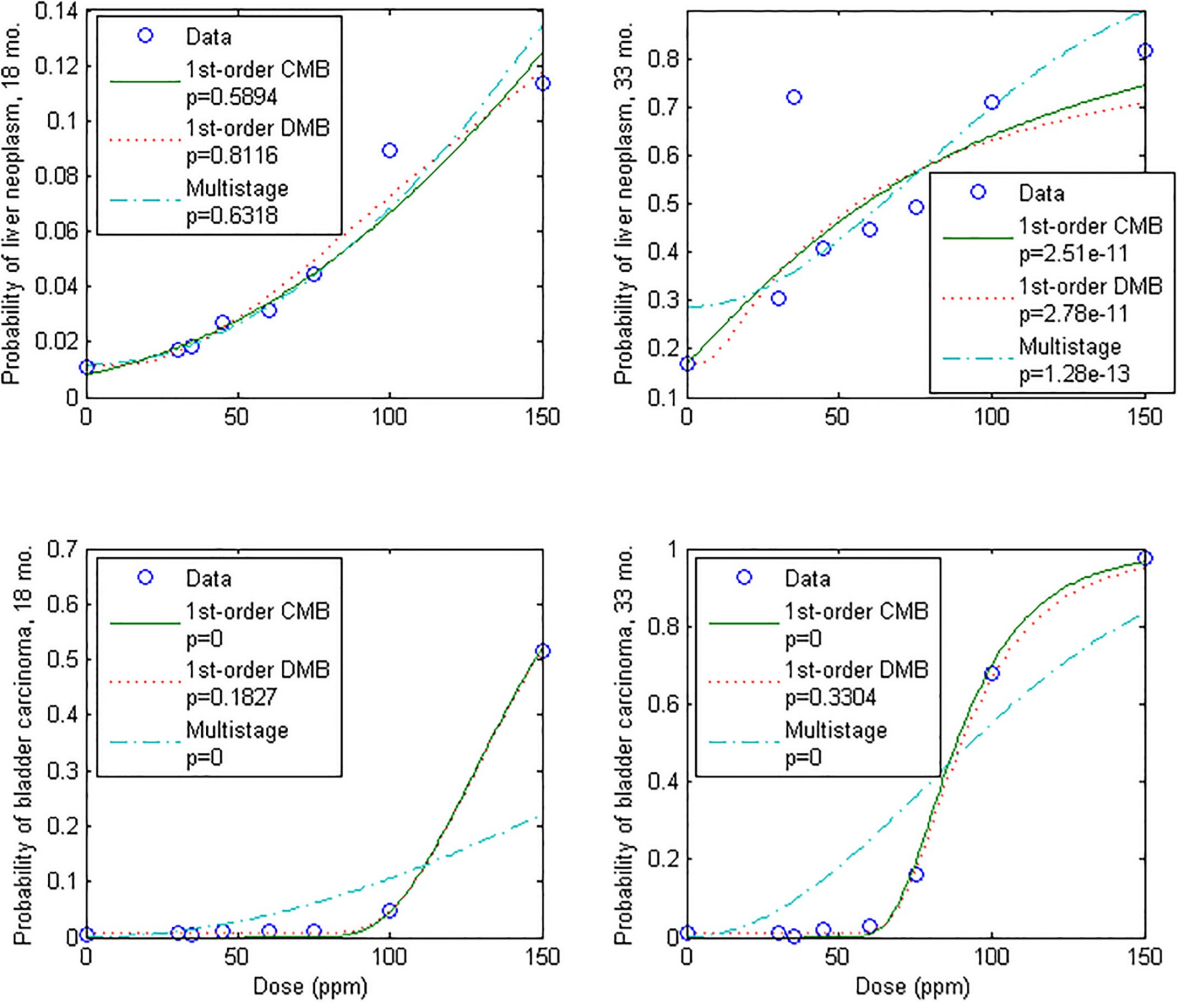
First-order/dissimilar-mode background (DMB), first-order/common-mode background (CMB), and two-stage DRFs versus data on 2-acetylaminofluorene-induced liver neoplasms and bladder carcinomas at 18 and 33 months, in mice. (p = 0 denotes a *p*-value below machine precision).

Results are shown in Fig 5 and Table 1. Again, the first-order model was associated with the highest *p*-values for all datasets. In addition, the fit of the first-order model was accepted for all datasets by GOF analysis (*p* ≥ 0.05), except for the data on liver neoplasms at 33 months which include an apparent extreme outlier (at 35 ppm) and which therefore did not fit any models. Although the much higher *p*-values for the first-order DMB model, when fitted to the 18 and 33-month bladder carcinoma data, appear anomalous when compared with the *p*-values for the first-order CMB models, which appear almost identical visually and mathematically, the result is factual. That is, the high *p*-values for the first-order DMB model represent sharp minimums in the log-likelihood function at the specific values of the background parameter, *c*, given in Table 1. Such a minimum could not be reproduced using the other models. Thus, it appears that in this case the χ^*2*^-test is able to clearly distinguish between the models and their treatment of background stress, due to the extremely large sample numbers. In general, the one- and two-hit multistage models could not be reasonably fitted to the bladder carcinoma data, which were much more sigmoidal than the model could represent. This lack of flexibility of the multistage model was confirmed in convergence checks, in which fit to the 18-month data could not be improved manually, and visual fit of 33-month data could be improved (somewhat) only at the expense of the *p*-value.

**Fig 6:**
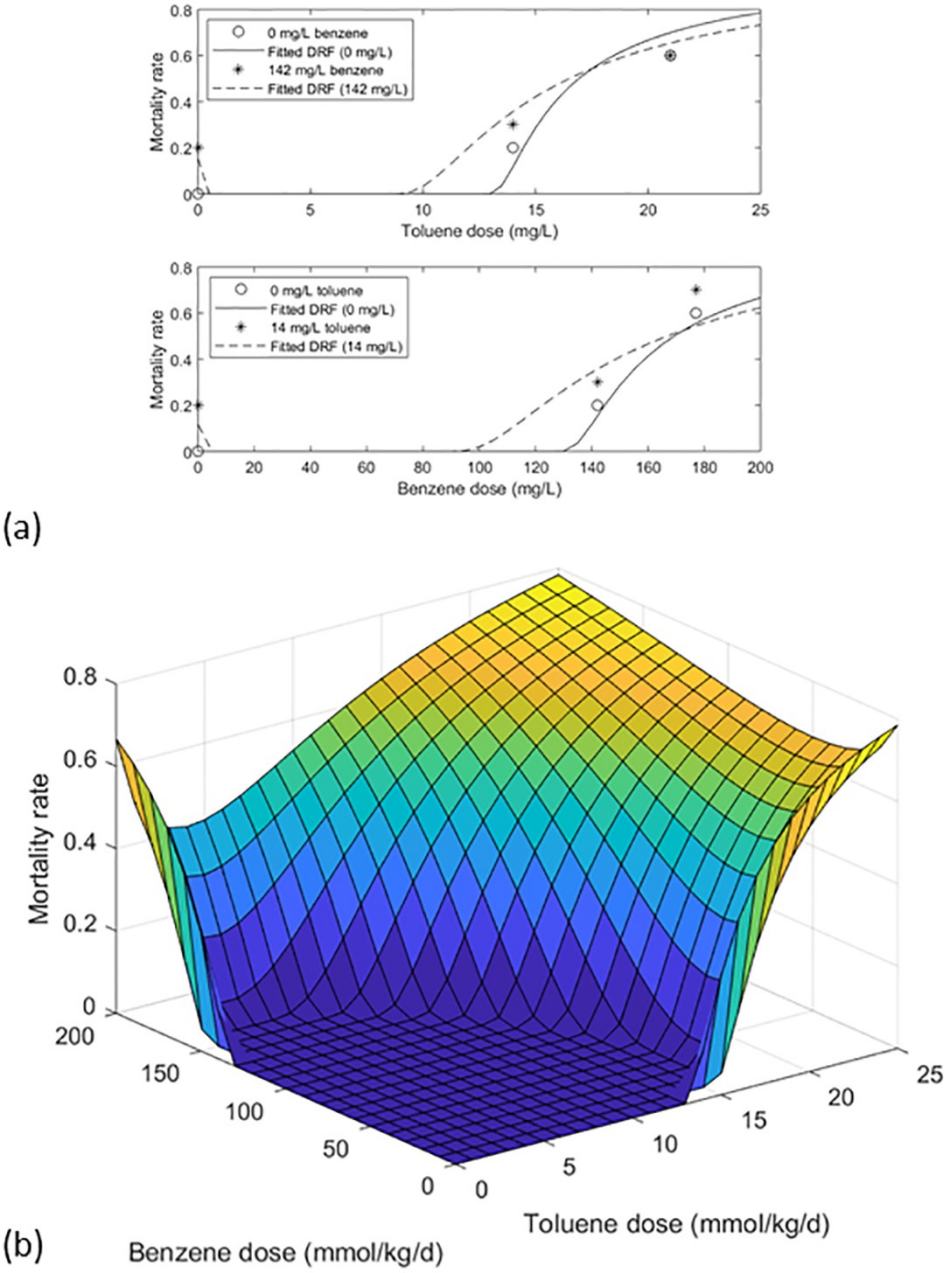
First-order common mode DRF fitted to data on medaka mortality following 96 hours of embryonic exposure to benzene/toluene mixtures. (a) mortality versus toluene dose, with and without 142 mmol/kg/d benzene, and mortality versus benzene dose, with and without 14 mmol/kg/d toluene; and (b) fitted DRF, showing suggested antagonism.

**Fig 7:**
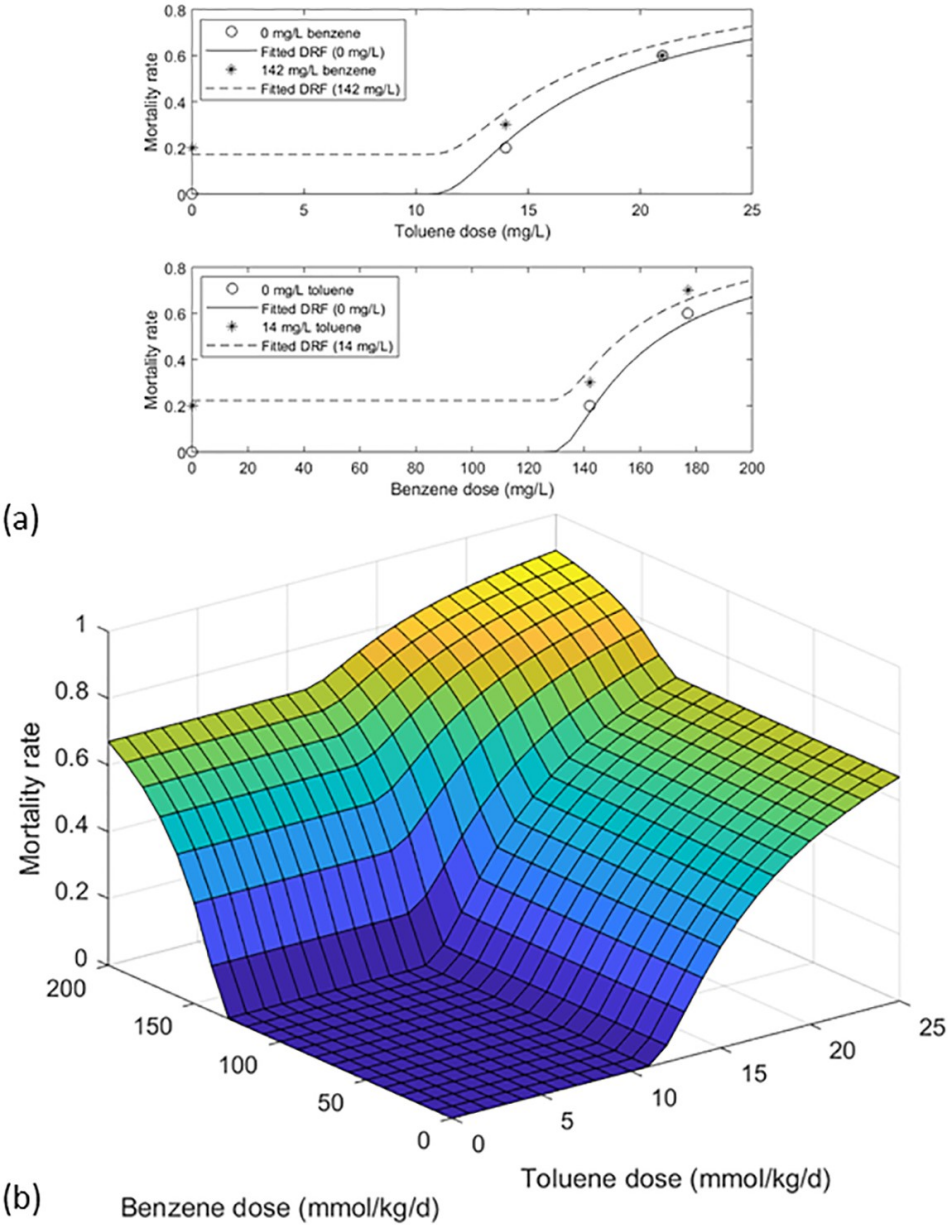
First-order dissimilar mode DRF fitted to the data of Fig 6. (a) mortality versus toluene dose, with and without 142 mmol/kg/d benzene, and mortality versus benzene dose, with and without 14 mmol/kg/d toluene; and (b) joint fitted DRF for mortality, assuming no interaction.

To allow demonstration of the models for mixture dose-response assessment, a dataset representing a full matrix of dose pairs, developed at least in part for examining mixture dose-response relationships, was provided by Teuschler, Thiyagarajah, and coworkers (S1 Table) [22]. Common- and dissimilar-mode first-order models, Eqs 2 and 7, were compared with this orthogonal multivariate data on mortality in Japanese medaka (*Oryzias latipes*) following 10-day embryonic exposure to binary mixtures of benzene and toluene (Table 1). Parameters of the first-order models were estimated by visual fitting of the 2-D curves shown in Figs 6(a) and 7(a). Subsequent to this work, similar fits to these data were obtained using a proposed gradient Markov chain Monte Carlo computational technique, presented previously [23].

Results of the first-order common mode model are shown in Fig 6 and Table 1, with the non-zero parameter *ξ*_*1*,*2*_ = -10.5 suggesting antagonism. Of note, the J-shaped dose-response relationship, which can be seen in one dimension for either chemical in the presence of the other on the response surface plot, can be seen to result from this antagonism. For example, as the concentration of toluene increases from zero in the presence of a constant 200 mmol/kg/d benzene, the antagonistic effect increases, so that net toxicity decreases before eventually increasing at higher doses of toluene. No significant difference was found between Eq 2 and the data (*n* = 8 doses; *α* = 0.05; *p* = 0.2825). Thus, results indicate that an observed J-shaped DRF may sometimes point to the presence of a second, possibly unrecognized, antagonistic, common-mode stressor, rather than to non-monotonic toxicity.

In Fig 7 and Table 1, the first-order dissimilar mode DRF, Eq 7, is fitted to the data of Fig 6. Interactions were not analyzed due to the lack of sufficient data to fit eight parameters of the bivariate model. Again, the fit was accepted (*n* = 8 doses; *α* = 0.05; *p* = 0.7804). While the somewhat improved fit suggests the possibility of dissimilar mechanisms, this improvement may also be explained by the higher parameterization of the general first-order model (in this case, 6 parameters versus 5 for the common mode model).

As a comparison of Eqs 2 and 4 with competitive DRFs, a general alternative would be the log-linear form of the generalized linear model [52]. However, that model could be written as Eq 2 with *η* = 1, and in fact the fitted values of *η*, *η*_*1*_, and *η*_*2*_ found for these data were all constant at unity. Therefore, for these data the log-linear model is equivalent to Eq 2 in terms of fit and form.

## Discussion

As mentioned previously, the single-chemical and mixture DRFs presented in Eqs 1–6 can be considered special cases of Eq 4, termed here the emergent DRF. This DRF is derived based on a general mechanism by which outcomes have been argued to develop across scales [32], accounting for important cause size correlation along a trajectory, and producing a distribution of outcome sizes previously demonstrated for illness severity [18] and other physical and biological outcomes [35,39,40]. Because no biological mechanisms specific to particular toxicants were assumed as bases for the resulting emergent DRF, it may be useful, for example, as an alternative to the lognormal and multistage dose-response models for carcinogens, noncarcinogens, and other non-microbial stressors. However, uncertainty in the parameters of any DRF, such as related to extrapolation from (possibly genetically homogeneous) lab animals to humans, and experimental variability, must generally be accounted for [53–55].

Dose-response uncertainty has most often been accounted for by computing confidence bounds [50,53,56,57]. Alternatively, a DRF which is unconditional with respect to parameter uncertainties and hence somewhat “broader” such that the dose associated with a policy-derived acceptable response level is lower, can obtained by the theorem of total probability [23,58,59]. (That is, the *unconditional* DRF is obtained by multiplying the DRF by the univariate or joint distribution of parameter uncertainty, and integrating over the parameter range.) In either case, Bayesian implementation allows use of non-traditional input information such as HTS data. In fact, a tiered Bayesian strategy for characterizing population variability, using *in vitro* data as Bayesian prior information to reduce reliance on animal data, has recently been proposed and evaluated [15]. In all of these approaches, the underlying form of the distribution of population variability is important for uncertainty analysis and extrapolation beyond the range of the data, and as a template for integrating multiple input information types.

The emergent DRF model may be useful as an indicator of toxicological mechanisms, when data are sufficient. As an example, the proposed common- and dissimilar-mode models were previously fitted [23] to published data [60] on cholangiocarcinoma following exposure to mixtures of PCB 126 and PCB 153. Effects of both PCBs are well-known to include cancer and other health impairments [61,62]. Although the common-mode model contained only four parameters, whereas the dissimilar-mode model had five, the common-mode model produced a higher GOF *p*-value (0.8348 versus 0.1925). While data were insufficient to distinguish between the models, and neither model could be rejected, such a result based on more data might suggest some commonality in the toxicity of these structurally-similar compounds, though PCB 126 is biochemically dioxin-like whereas PCB 153 is not. In addition, the common-mode model suggested significant synergism between the two toxicants (1/ξ_1,2_ was on the same order as ξ_1_ and ξ_2_), consistent with reports of pharmacokinetic interactions between PCB 153 and dioxin-like compounds including PCB 126 [63].

Based on current results, the following conclusions can be drawn:

1. A general emergent DRF is derived theoretically, initially demonstrated in preference to the lognormal and multistage models for all cancer and non-cancer datasets analyzed, and shown to fit all datasets except one with extreme outlier included. These results are consistent with previously published demonstration of the model versus dose-response data on crocidolite, and on benzene-toluene and PCB 126-PCB 153 mixtures [23,24];
2. Subject to continued verification, e.g. versus animal and PBPK data, the derived common- and dissimilar-mode mixture models allow the assessment of the cumulative risk of chronic stressors, potentially including chemical, environmental, occupational, lifestyle, economic, and other factors, that can be expressed in terms of a homogeneous (e.g., non-microbial) “dose;”
3. Because of the scale-inclusive and general nature of the model, extrapolation across scales in terms of dose and physiological processes, and applicability across toxicological pathways and endpoints, are theoretically supported for generalized cases;
4. Though the basic univariate form of the emergent DRF has only two parameters, like the lognormal and the single-hit cancer models, it can assume either monotonic form like the single-hit, as may be plausible for non-threshold toxicants, or sigmoidal form like the lognormal, as may be expected particularly for threshold toxicants;
5. The common-mode emergent mixture DRF is a generalization of the log-linear form of the generalized linear model; and
6. Results of the mixture dose-response analysis presented illustrate how a difference in toxic mode of action affects the joint DRF, and particularly how a chemical having a typical monotonic DRF can present a J-shaped DRF when a second, antagonistic, common-mode stressor is present in the mixture.

Though the generality of the emergent model may sacrifice some specificity, generality may be important in toxicity screening, low-dose extrapolation, and the identification of disease drivers, when uncertainty is accounted for explicitly [23,24]. Therefore, it is suggested that:

1. The model be further demonstrated versus traditional and new data types, due to the limitations of biological data and the potential for stressor interactions;
2. The model be tested as an alternative for traditional dose-response assessment of chronic chemical and other health stressors, and mixtures having common endpoint, and in Bayesian and other non-traditional assessments potentially using HTS, multi-tissue co-culture, multi-organ chip, and animal data;
3. To address parameter estimation for multicomponent mixtures, development of new fitting algorithms should be continued, e.g. building on computer programs published previously for the bivariate emergent DRFs and corresponding predictive Bayesian versions [23];
4. The theoretical basis of the emergent DRF may be considered in using HTS and other biomarker data to estimate parameters, and perhaps vice versa. For example, HTS data may be useful in estimating the distributions of illness cause sizes from which the DRF derives, likely via predominantly first-order kinetics. Also, selected biomarker data may represent intermediate medical status along an MOA, though such status may be below the tipping point for irreversible impairment, and the first-order severity model applies to such intermediate status as well as to final severity. Thus, for example, the value of the parameter, *η*, of the DRF, which is directly related theoretically to the analogous parameter (theoretically constant with dose) of the illness severity distributions, might reasonably be inferred to be smaller than the value of *η* found for the Weibull distribution of severity of the preceding biomarker, because the inverse value of *η* indicates the extent of first-order compounding to that point along an MOA.

## Supporting information

S1 Table Published dose-response data: Chloroform, bromate, and benzene/toluene mixture. (DOCX)

S2 Table Published dose-response data, 2-acetylaminofluorene in mice [20]. (DOCX)
