# Supplementary material for: A general dose-response relationship for chronic chemical and other health stressors and mixtures based on an emergent illness severity model
Source: PLoS One. 2019 Feb 15;14(2):e0211780. doi: 10.1371/journal.pone.0211780 (PMC6377108; doi:10.1371/journal.pone.0211780)
Supplement: S1 Table — (DOCX) [file pone.0211780.s001.docx]

**S1 Table. Published Dose-Response Data: Chloroform, Bromate, and Benzene/Toluene Mixture**

| Chloroform [19] | | Bromate [21] | | Benzene dose (mmol/kg-day) | Toluene dose (mmol/kg-day) | Deaths/  Total [22] |
| --- | --- | --- | --- | --- | --- | --- |
| Dose (mmol/kg-day) | Mild liver necrosis/  total | Dose (mg BrO_3_^-^/ kg-day) | Dysplastic focia/  total |  |  |  |
| 0 | 1/12 | 0 | 0/19 | 0 | 0 | 0/10 |
| 0.152 | 0/13 | 0.7 | 1/19 | 142 | 0 | 2/10 |
| 0.305 | 8/15 | 1.3 | 5/20 | 177 | 0 | 6/10 |
| 0.76 | 14/15 | 2.5 | 6/24 | 0 | 14 | 2/10 |
| 1.52 | 13/13 | 5.6 | 12/24 | 142 | 14 | 3/10 |
| 3.05 | 12/12 | 12.3 | 19/20 | 177 | 14 | 7/10 |
|  |  | 33 | 19/20 | 0 | 21 | 6/10 |
|  |  |  |  | 142 | 21 | 6/10 |
