# Supplementary material for: A general dose-response relationship for chronic chemical and other health stressors and mixtures based on an emergent illness severity model
Source: PLoS One. 2019 Feb 15;14(2):e0211780. doi: 10.1371/journal.pone.0211780 (PMC6377108; doi:10.1371/journal.pone.0211780)
Supplement: S2 Table — (DOCX) [file pone.0211780.s002.docx]

**S2 Table. Published dose-response data, 2-acetylaminofluorene in mice**

| Dose | Response (no. positive/total no. tested) | | | |
| --- | --- | --- | --- | --- |
| (ppm) | Liver neoplasms  18 months | Liver neoplasms  33 months | Bladder carcinomas  18 months | Bladder carcinomas  33 months |
| 0 | 6/555 | 17/100 | 2/553 | 1/101 |
| 30 | 34/2014 | 135/445 | 14/2008 | 5/443 |
| 35 | 20/1102 | 72/100 | 4/1103 | 0/200 |
| 45 | 15/550 | 42/103 | 6/545 | 2/103 |
| 60 | 13/411 | 30/67 | 5/410 | 2/66 |
| 75 | 17/382 | 37/75 | 4/382 | 12/75 |
| 100 | 19/213 | 22/31 | 10/213 | 21/31 |
| 150 | 24/211 | 9/11 | 107/207 | 11/11 |
